# Balancing perceptions of targeting: An investigation of political microtargeting transparency through a calculus approach

**DOI:** 10.1371/journal.pone.0295329

**Published:** 2023-12-07

**Authors:** Martin-Pieter Jansen, Nicole C. Krämer

**Affiliations:** 1 Social Psychology: Media and Communication, University of Duisburg-Essen, Duisburg, Germany; 2 Research Center Trustworthy Data Science and Security, Dortmund, Germany; Roma Tre University: Universita degli Studi Roma Tre, ITALY

## Abstract

Over the last few years, political advertisers have moved with their audiences: to social media platforms. Advertisers on these platforms aim to persuade voters by sending messages tailored to them based on their own data: political microtargeting (PMT). A considerable problem with PMT is that users are often unaware that they are being targeted, while current transparency advances do not seem to suffice in informing users. However, increasing transparency may have consequences on users’ privacy perceptions. Thus, the current work investigates whether disclosures, as a measure to increase transparency, increase users’ recognition of a microtargeted ad, and subsequently what this means for their perceived benefits, privacy concerns, and their likelihood of engaging in privacy protection behavior, based on the privacy calculus. In a preregistered online one-factorial between-subjects experiment (*N* = 450) we exposed participants to either an Instagram post containing a currently used disclosure or a more salient disclosure. Our results show that exposure to this disclosure increases recognition of the ad being microtargeted, and that this relates to perceived benefits but not privacy concerns. However, the results show that users’ privacy concerns are related to their increased privacy protection behavior. Finally, we found that over four-fifths of our participants who were exposed to the more salient disclosure recalled it correctly.

## Introduction

A by-product of people’s use of social networking sites (SNS) is the data they leave behind. These digital breadcrumbs, left behind through liking posts, commenting on videos, and simply interacting with or viewing content, are valuable to advertisers and political consultancy agencies. Through these breadcrumbs or data points, they build profiles of users and allocate them into small groups while narrowly targeting them with specific messages that are developed to resonate most effectively within these groups, a practice commonly titled *microtargeting* or in a political context *political microtargeting* (PMT) [1–4].

PMT can not only be used to persuade voters, but also to discourage political participation, potentially negatively affecting voter turnout, which damages the democratic process [5, 6]. Moreover, sending messages to parts of the public while nobody else, but that part of the public knows of the existence of that message avoids scrutiny in the democratic process as well, harming it instead [7]. Besides, campaigns could base their actions on personal data, while users are not aware of the information used to target them, if they are even aware that they are being targeted at all, potentially opening the door for voter manipulation [8]. However, PMT also provides opportunities for voter mobilization [4], increasing political interest [8], and supporting voters to access relevant information [9]. Recent work [10] finds both positive and negative effects and speaks of a paradox of PMT, where the technique might benefit individuals through usefulness but might be harmful for society at large.

In the most commonly known example of political targeting British political consultancy firm, *Cambridge Analytica* presumably gathered and used data from more than 50 million Facebook users to form psychological profiles and target users with messages that would persuade them as strongly as possible [11]. This allegedly contributed to Trump’s presidential victory and the Leave campaign’s success in the Brexit referendum. However, critics claim that users’ privacy is at risk here and that this is mostly without their informed consent [12–14].

A major problem with PMT is that users do not recognize the targeted, persuasive attempts that campaigning agencies and parties use to try to affect them [2, 15]. A potential solution to this problem is the use of disclosure labels. Platforms such as Facebook, Instagram, and Twitter have been using these labels to be transparent and to provide users with a tool to distinguish sponsored posts or advertisements from regular content on the platform. Marketing and advertising research has investigated these labels for a long time [16–18]. In the context of PMT, however, research on the labels is upcoming but still scarce [1, 2, 19, 20]. Disclosures have been found to increase users’ knowledge and perceptions of persuasive attempts, which can be investigated through the Persuasion Knowledge Model [21], or in a PMT context through targeting knowledge, which is based on the Persuasion Knowledge Model and adapted to the context of targeting [1].

One of the theoretical frameworks that helps us understand users’ privacy perception is the privacy calculus by Culnan and Armstrong [22]. The privacy calculus assumes that users’ behavior regarding their privacy is influenced by their perceived benefits and perceived costs or risks. If users are aware of targeting that occurs, this might lead to different perceptions concerning their online privacy, as has been found in research on advertising personalization [23]. To the best of our knowledge, an empirical investigation of the privacy calculus in the sphere of PMT has not yet been conducted.

Prior research on PMT has mainly focused on Meta’s Facebook as a platform [1, 2, 20]. While the company’s other major platform, Instagram, has grown faster than Facebook [24], an empirical investigation of the use of targeting disclosures on Instagram has not been conducted. Since the platforms have different focuses on content and users might have different needs to fulfill while using them, there might be different effects of these disclosures.

Therefore, the current study investigates two different disclosures: a regular disclosure that is currently used on Instagram showing that content is sponsored and a more elaborate and salient disclosure that is a partial replica of the disclosures that were used on Instagram to inform users about potential fake news regarding COVID-19 information. We investigate the effects of these two disclosures on users targeting knowledge. Moreover, we will focus on users’ privacy perceptions by investigating a micro-level perspective on the privacy calculus, where perceived benefits and privacy concerns potentially lead to privacy protection behavior.

## Theoretical background

One of the major problems regarding PMT is the presumed black box, where neither laypersons, journalists, nor researchers know exactly what political consultancy firms do to microtarget users and how and which data are used. Although this black box makes it difficult to research PMT, it also makes it practically impossible to estimate the effects of campaigns run by firms and parties. Because the lack of transparency and information about the models used makes it difficult to focus on the sender side of PMT, one solution could lie within the user side of this way of campaigning. Regulatory bodies are starting to implement regulations to provide users with appropriate transparency measures. For example, the EU Digital Services Act states that targeted information and advertisements should include information on when and on whose behalf, content is displayed. Simultaneously, these measures should go beyond that and include background information about individualized data used to target users and parameters for those data points [25, 26].

### Disclosures

The way the EU Digital Services Act aims to have platforms implement disclosures is the first time that targeting disclosures have been initiated in Germany. Currently, the ‘fake news’ bill, that also includes targeting transparency on platforms, in Brazil is being reviewed in congress [27]. Within the EU, earlier regulations were proposed in Ireland [28] and adopted in France [29, 30]. Earlier versions of disclosures only aimed to inform users that content is sponsored (e.g., YouTube, Instagram, and Facebook), and in some cases who the party paying for the sponsored content is (e.g., Twitter and political advertisements on Facebook). Research on the application of disclosures in a microtargeting context has emerged for a couple of years and shows mixed results regarding the recall of disclosures and their effectiveness in helping users recognize political advertising [1, 2, 20]. Nonetheless, one of the main implications of disclosures is that the prominence, position, and degree to which they stand out in contrast to a user’s timeline are important [16, 31]. Furthermore, the combination of text and a symbol leads to the highest visual fixation [17]. While being more prominent and therefore standing out compared to regular content, disclosures lead to higher levels of attention and, in turn, to better perception of the disclosed information, and thus contribute to transparency [1, 2]. The status quo for platforms seems to be a short gray sentence stating that content is sponsored or sponsored, and by whom it is paid for. However, this status quo seems to be insufficient in informing users, thus contributing to transparency, as most of the disclosures that are currently used on platforms lead to low levels of recallment [1, 19, 20].

Since 2021, differently designed disclosures have been used on various platforms. During the COVID-19 pandemic, both Instagram and Facebook took measures to counter the spread of misinformation and to inform users. One of these measures was the use of disclosures on posts and stories (disappearing posts) on the platform that guided users with both information to directly inform them that information could be false, or was not yet proven, and a link to Instagram’s ‘COVID-19 Information Center’, where users could find credible information about COVID-19 in general [32, 33].

To the best of our knowledge, these new disclosures have not been investigated in a scientific setting, nor are there results or information publicly available from Meta. It seems promising that the platform experiments with different types of disclosures. From a business perspective, it would make sense to not label advertisements on platforms as large as the platforms did with the COVID-19 information because selling room for advertisements is a major part of their business models. However, since an increasing number of legislations seem to be upcoming (e.g., the EU Digital Services Act), using these already existing disclosures might be a great solution for platforms to oblige to these legislations. Therefore, the current work will use Instagram’s existing sponsored disclosure as a control condition while also going beyond that and consequently investigate disclosures that inform users about targeting.

### Targeting knowledge

Persuasion is something that users are exposed to every single day, both in the ‘real world’ and on social networking sites. To cope with persuasive messages, users develop beliefs and knowledge about persuasion based on their previous experiences with and exposure to advertisements: *persuasion knowledge* [21]. Existing work on disclosures shows that if a message contains a disclosure, it is more likely that users’ persuasion knowledge will be activated compared to when a message does not contain a disclosure, in both research on regular advertising [34, 35] and microtargeting [2, 36]. However, as persuasive tactics evolve and move to places where the potential targets of these messages are, online, it is deemed important to also evolve the measurement instruments to reflect the specific context. In recent work on PMT, Binder and colleagues [1] developed a scale for *targeting knowledge* based on earlier work on personalization and the persuasion knowledge model [21, 37, 38]. For this construct, the authors use the following definition: “Individuals’ beliefs of agents’ use of their online data to tailor messages to recipients” [1]. The authors also underline the importance of the construct covering not only the recognition of targeted advertisements, which is usually lower than that of a regular advertisement [39], but also users’ perceptions of targeted messages, as the way the message is designed, constructed, and delivered, in line with the persuasion knowledge model [21]. Binder and colleagues found good reliability for their newly developed measure [1].

Therefore, this study builds on this new concept and attempts to empirically investigate it with a more prominent, salient, and potentially more attention-grabbing disclosure, including both a textual aspect and a symbol informing users that the disclosure contains additional information, which has been found to improve transparency regarding the persuasive or targeted nature of the message [2, 20, 40]. Taking this into account, we expect that a more salient and prominent disclosure increases users’ recognition of targeting practices:

H_1_: Exposure to a more prominent targeting disclosure, compared to Instagram’s regular sponsored disclosure increases targeting knowledge.

### Privacy

One of the problems with using data for advertising purposes, as done in microtargeting, is that it crosses boundaries regarding users’ privacy on SNS. An example of this is work that shows that online behavioral advertising (OBA), which could be viewed as the precursor of PMT, is often perceived as a privacy risk and intrusive [23]. The same work, however, also sheds light on the perceived benefits of OBA and shows that personal relevance, added advertising value, and economic benefits are the top three perceived benefits in their US national sample. One framework that helps us understand online privacy and users’ perceptions of it is Culnan and Armstrong’s privacy calculus theory [22]. The privacy calculus theory states that people weigh privacy costs and benefits before they disclose personal information and are more likely to disclose this information if the benefits are at least balanced (if not greater than) the privacy costs [41]. Regarding privacy on social media, this could be seen as the subjective experience of privacy, as described in the model by Trepte [42]. According to this framework, the user experiences access that results from their usage goals on the one hand and the social media boundary conditions and privacy mechanisms on the other. Subsequently, these privacy perceptions lead to different forms of privacy regulation behaviors, such as altering self-disclosure and control by restricting access to information [42]. Moreover, in empirical work, Dienlin and Metzger [43] showed that in the context of SNS, their extended privacy calculus, including users’ self-withdrawal behaviors and privacy self-efficacy, holds true. However, regarding self-disclosure, the benefits outweigh privacy concerns, while in the case of self-withdrawal, privacy concerns outweigh both self-efficacy and benefits. Other work shows that, in the case of app usage concerning a COVID-19 warning app, both privacy concerns and perceived benefits predict app usage [44]. Although there have been other studies that provided a theoretical framework [45], investigating aspects of privacy regarding microtargeting over time [13, 46], and with an adolescent sample [47], an empirical investigation of the privacy calculus in a microtargeting context has not yet been conducted.

In the context of microtargeting, we propose that the calculated rational approach grounded in the privacy calculus can explain users’ behavior regarding their online privacy. Users might experience privacy concerns through the use of their personal and behavioral data while also having concerns regarding data protection [1, 13, 23]. Simultaneously, users may experience higher usefulness by receiving political advertisements that match their personal preferences or interests [1, 48]. These experiences and perceptions can coexist and lead to changes in attitudes towards PMT techniques and users’ privacy protection behavior. Engaging in more protection behavior could protect them against data usage by platforms and advertisers on the one hand, while on the other hand, engaging less in this behavior might give platforms and advertisers more information and data to work with, potentially leading to better targeted ads and thus higher usefulness. Thus, we aim to investigate political microtargeting through the propositions of the privacy calculus theory while analyzing the perceived benefits and privacy concerns users experience concerning PMT. Subsequently, we scrutinize whether these perceptions and concerns lead to differences in privacy protection behavior.

### Perceived benefits of microtargeting

The benefits side of the calculus rationale can be described as the advantages that users receive in exchange for their online data, both self-disclosed and gathered through behavior. In existing work, the benefits of PMT are explained primarily on a societal (or macro) level, and the overall consensus seems to be rather negative. However, PMT may also be beneficial. The use of the technique can, for instance, activate potential voters who are usually deemed to have a lower propensity to vote by reaching out to them in personally relevant ways, with messages that are personally relevant as well [4], which has the potential to strengthen general political interest [8, 10]. Nevertheless, these benefits occur at the macro level. At a personal level, personalization of content leads to higher levels of attention, more accurate recall, and more positive evaluations of content [49]. Other work does not find that higher levels of personalization lead to higher levels of perceived relevance, but this could be a result of the fact that SNS users might not be aware that so much information is gathered concerning them and is used to tailor advertisements to them [50]. Furthermore, in a study on the AdChoices Icon, which could be seen as a version of a disclosure, and personalization Brinson and Eastin [51] found that when consumers, due to a disclosure, recognize an advertisement that contains personalization, they have more favorable attitudes towards that ad.

Moreover, the personal benefits of PMT might lead to societal benefits when a reduction in time and cognitive effort in obtaining information and higher content relevance mobilizes individuals to vote [2]. Furthermore, previous work on online behavioral advertising found that the technique can narrow down alternative solutions to the most relevant and helpful information [9]. Similarly, Zarouali and colleagues [4] conclude that PMT can serve as an effective way to provide relevant information to citizens on the issues they really care about, which could lead them to be more informed and knowledgeable about these issues. Moreover, personal relevance could lead to higher motivation to process information, which could subsequently lead to a more central attitude change, as described in the Elaboration Likelihood Model (ELM) [52]. Taking the macro-level benefits into account, this study empirically investigates the micro- or individual benefits of PMT, and we expect that users who recognize targeted advertisements experience higher levels of benefit perceptions:

H_2_: Targeting knowledge is positively related to perceived benefits

### Privacy concerns

As stated above, the overall consensus on PMT and its effect on society appears more negative. Multiple authors underline their concerns on PMT potentially: discouraging political participation [5], decreasing scrutiny in the democratic process by leaving out certain target groups [7], enlarging the gap in representation in governments [6], and manipulating voters by targeting users without their knowledge or consent [8]. These concerns, however, are all on a societal level, meaning that they potentially harm individual users but are not necessarily concerns users have if they are aware of a message being targeted at them.

On an individual level, users perceived privacy concerns regarding personalization and microtargeting; for example, an advertisement is perceived as a privacy risk and intrusive [23]. Other work shows that higher levels of personalization lead to higher perceived *creepiness* of advertisements [50]. Recent work validating a scale on perceived surveillance concerning personalization effects found that users experience creepiness, concerns about surveillance, perceptions of privacy risks overall, and privacy concerns [53]. In addition, other work found that in the case of data-driven OBA, persuasion knowledge, on which the concept of targeting knowledge is built, positively affects privacy risks, which could be considered as the cost side of the privacy calculus theory [54]. Dobber and colleagues [13] found that privacy concerns regarding PMT lead to more negative attitudes towards the technique and reversibly a higher attitude towards the technique leads to a decrease in privacy concerns. Taking this into account, we propose that on an individual or micro level, users who recognize targeting advertisements feel that the technique violates their privacy standards:

H_3_: Targeting knowledge is positively related to privacy concerns

### Attitude towards the platform

While making users aware of the targeting practices taking place on SNS might lead them to change their perceptions regarding those practices, user attitudes might also influence this relation. In an earlier study on Facebook, Debatin and colleagues [55] found that while users did recognize the potential privacy issues of the platform, they simultaneously uploaded large amounts of personal information, and that this behavior may be explained through high levels of gratifications of using the platform. Furthermore, the mood congruency hypothesis assumes that a recipient’s mood state may influence the associations generated during exposure to a message, leading to more positive elaboration of the content or more positive reactions to peripheral cues [56]. Other work shows that in the domain of intrapersonal communication, the medium used to send a message affects its persuasiveness [57]. Moreover, in work on personalized advertisements, De Keyzer and colleagues [50] found that the source type can mediate the effect of perceived relevance on source attitude. Furthermore, in work on personalization and the privacy calculus, Hayes and colleagues [58] found that the consumer-brand relationship has a positive moderating effect on the benefits side of the privacy calculus, meaning that for users with a stronger consumer-brand relationship, the effect of perceived benefits on the value of information is larger than for users with a weaker consumer-brand relationship. In addition, in the case of adolescents, previous work finds that privacy perception and data protection are positively affected by social media activity [59], meaning that users who find social media use important have higher levels of privacy perception and find data protection more important.

In this study, we scrutinize if the attitude users have towards the platform (Instagram) beforehand, leads to a difference in the relations of targeting knowledge, perceived benefits, and privacy concerns regarding PMT. More specifically, we propose that for users who have a more positive attitude towards Instagram, the relationship between targeting knowledge and perceived benefits is stronger, meaning that if a user is happy with the platform and likes to use it, the microtargeted advertisement might be perceived as more beneficial. Similarly, we propose that for users who have a more negative attitude towards Instagram, the relationship between targeting knowledge and perceived benefits is weaker, meaning that if a user is not happy with the platform and, for instance, is already contemplating leaving it, the microtargeted advertisement might be perceived as less beneficial, just because the user is less happy with Instagram. Leading us to propose the following two moderation hypotheses:

H_4a_: The relation between targeting knowledge and perceived benefits is moderated by users’ attitude towards the platform
H_4b_: The relation between targeting knowledge and privacy concerns is moderated by users’ attitude towards the platform

### Privacy protection behavior

As mentioned, the rationale described in the privacy calculus leads users to engage in more or less self-disclosure on social media. However, recent work provides us with a different outcome of the calculation between privacy costs and benefit perceptions: privacy protection behavior [60]. Examples of privacy protection behavior range from altering the privacy settings on a platform or using software to disguise oneself to deregistering from the platform altogether. We deem privacy protection behavior a more fitting outcome measure in the case of PMT because users are not always aware of the information that is used to target them, which is not always self-disclosed. Earlier work found that perceived risks lead to a lower intention to self-disclose and a higher desire for protection, which in turn leads to an intention to use a tool to protect oneself online [60]. Moreover, perceived manipulative intent has been found to increase users’ privacy behavior [1]. Additionally, higher levels of privacy concerns lead to higher intentions to withdraw information from Facebook and lower intentions to disclose information on the platform as well [43]. Besides, Büchi and colleagues [61] found that when people feel that their online privacy has been violated, they implement greater privacy protection.

Furthermore, other work on the personalization of advertisements suggests that the perceived costs (or privacy concerns) outweigh the perceived benefits, which we propose leads to engaging in more privacy protection behavior [50]. In a microtargeting context, recent work shows that privacy concerns lead to more privacy protection behavior, but interestingly, it does not lead to users applying ad blockers to block the advertisement [46]. This is in line with other work, showing that in the case of regular online advertising, opt-out rates via AdChoices (a platform that uses cookies to tailor ads to website users) are 0.26% in the European Union and 0.24% in Germany [62]. This could raise the question of whether there are boundaries for privacy protection behavior, meaning that users will engage in it if the measure is not too technical or too much of a procedure to take.

Conversely to what is explained above, we expect that users that have higher levels of perceived benefits regarding PMT, will engage less in privacy protection behavior, which is in line with a lot of work that shows that perceived benefits lead to more self-disclosure. We recognize that even though the outcome measure might be less fitting for this work, the mechanism behind it might still hold true. For instance, someone who is happy with a tailored ad will be less motivated to disable the ad by altering their advertising preferences on social media. In this study, we investigate privacy protection behavior as the outcome from a privacy calculus perspective on PMT. We propose that perceived benefits are negatively related to privacy protection behavior, meaning that users who perceive high levels of benefits of PMT intend to take fewer precautions to protect their privacy online. Conversely, we propose that privacy concerns are positively related to privacy protection behavior, meaning that users who perceive high levels of privacy concerns concerning PMT intend to engage in more behavior to protect their online privacy:

H_5_: Perceived benefits are negatively related to intended privacy protection behavior
H_6_: Privacy concerns are positively related to intended privacy protection behavior

### Algorithmic user agency

In addition to investigating privacy protection behavior, we propose an exploratory outcome for users who experience the benefits of personalization or targeting. Users’ social media feed is filled with content, content from people and companies they follow, but also with content that they are more likely to engage with disregarding the sender. This content is usually targeted at them through an algorithm that knows what they previously engaged with, look at for a longer time, or scrolled back to.

Nevertheless, there is work that shows that users are not as surrendered to algorithms as one might think. In an exploratory study, Kapsch [63] found that some users influence what content they see by interacting with profiles, liking posts, commenting, or even texting via direct messages (DM): *algorithmic user agency*. Users who exercise these techniques try to gain autonomy by actively showing the algorithms of social platforms what they like, which potentially is a proxy for their willingness to actively use algorithms to their advantage. To the best of our knowledge, there has not been work discussing this concept concerning PMT. Using this construct, we aim to better understand users’ behavior that is intended to inform the algorithm about their preferences by consciously interacting with content in order to receive recommended content that is tailored better in the future. In the case of PMT, this behavior could lead to even more fitting targeting because the user is actively feeding the algorithm information about their personal preferences. This behavior can be exemplified by liking a cat video on Instagram to see more cat videos in the future. As an exploratory research question, we aim to investigate whether users’ perceived benefits of PMT relates to these users trying to influence the algorithm by employing algorithmic agency:

RQ_1_: What is the potential relation between users’ perceived benefits and behavior that would lead to users actively interacting with certain content: algorithmic user agency?

## Method

This study was approved by the ethics committee of the University of Duisburg-Essen (approval number 2211SPJM9646). We preregistered this study before collecting data: [link deleted for peer-review]. All participants agreed with our online informed consent form by checking a box on the page showing the consent form before participation; otherwise, participation was not possible, and participants were redirected to the website of our panel provider (Prolific). Supplementary materials and our measures are publicly accessible on OSF: https://osf.io/2rbqu/?view_only=7923aff35ecd44caa90f7c7603912e03.

### Design

To test our hypotheses, we conducted an online experiment using a factorial between-subjects design containing two groups. Participants were exposed to a political Instagram advertisement with a statement aligned with their beliefs. The advertisement contained either a sponsored disclosure, in line with the disclosures Instagram uses (‘Sponsored’) in the control condition, or a sponsored disclosure and a more salient targeting disclosure based on the false information disclosures the platform used during the COVID-19 pandemic. However, the disclosure was adapted to contain information about the post being targeted (‘This sponsored message is targeted at you based on your age, gender, and online behavior’) and was highlighted by a red square. Both stimuli can be found on OSF (https://osf.io/2rbqu/?view_only=7923aff35ecd44caa90f7c7603912e03). In total, we created four different Instagram ads, meaning that for every condition, we had pro- or anti-climate change regulations to fit the view of participants to simulate targeting (see Procedure). For both conditions, the number of likes, comments, and timestamp of posting (14 hours ago) were the same.

### Procedure

After providing informed consent, participants were briefed about the study and that the next page would include 10 political statements we needed them to either agree or disagree with. After answering the statements, which we used to investigate their point of view on climate change regulations, we briefed them again, this time about the next page containing an Instagram post which we asked them to closely look at, and that a ‘next’ button would appear after 15 seconds. After this briefing we exposed participants to our stimulus material containing a statement that was either pro- or anti-climate change regulations, fitting their earlier answers. Subsequently, we asked them questions regarding our variables and a manipulation check. We then asked the participants about their demographic information before we finally debriefed them and thanked them. The average completion time was five minutes.

### Sample

We recruited 464 adult German Instagram users through panel provider Prolific from December 13 to 16, 2022. We were unable to identify the participants individually in this study. Seven participants quit our questionnaire before completion, four participants failed our attention check, two participants timed out before completion, and one participant did not agree with our informed consent. Leaving us with a final sample of 450 participants that we included in our analyses. Within our sample, the age ranged from 18 to 70 years (M = 29.4, SD = 9.5). Of these participants, 220 identified as female, 223 as male, and 7 as diverse. Regarding education, most of our participants had a university entrance qualification (*n* = 150), 114 had a bachelor’s degree, and 89 had a master’s degree. Furthermore, 49 participants had an intermediate high school diploma, and 28 had an advanced technical college entrance diploma. Finally, 10 participants had a doctorate, five had a qualifying middle school diploma, and five participants responded that they had a different educational background. Randomization checks showed no differences between our two experimental groups regarding age (*F*(1, 448) = 0.45, *p* = .505), gender (*χ*2(2, *N* = 450) = 0.17, *p* = .921), or level of education (*χ*2(7, *N* = 450) = 8.37, *p* = .301).

### Power

Given the budget for this study, we were able to gather responses from 450 participants [64]. To determine our statistical power, we used R (version 4.1.2) [65] and the simsem package (Version 0.5–13) [66]. To establish our smallest effect size of interest (SESOI) [67], which could still be interpreted meaningfully given our current sample size and method, we employed various simulation analyses using the structure of our structural equation model (SEM). We set our power level at 90%, which is desirable at least [68], and kept our α level (α = .05) and sample size (*N* = 450) constant while testing various effect sizes. We ran 1,000 replications for our model. Finally, we set our SESOI at β = |.15| and did not interpret results with effect sizes smaller than this.

### Measures

All constructs used to measure our variables were tested for factor validity in confirmatory factor analyses (CFAs). The results of the CFAs and measures of reliability and internal consistency are shown in Table 1. We measured *targeting knowledge* through five items used validated in earlier research on PMT by [1] on a Likert scale ranging from 1 (= strongly disagree) to 7 (= strongly agree) (e.g., “The post is tailored to me”), one item “The post showed personalized advertising” decreased our indicators for a good fit in a CFA and thus was not included in our analyses. We measured *perceived benefits* through five items combining the most fitting items from Lavado-Nalvaiz and colleagues [69] that investigated the privacy calculus for smart home devices with items from Yang [70] investigating the privacy calculus for online behavioral advertising using a Likert scale ranging from 1 (= strongly disagree) to 7 (= strongly agree) (e.g., “The Instagram post is helpful”). Two items “Seeing an Instagram post that is targeted at me makes me happy” and “I know a targeted Instagram post fits and suits me” decreased our indicators for a good fit in a CFA and thus were not included in our analyses. We measured *privacy concerns* using a scale that has been used in recent microtargeting research by Dobber et al. [13], consisting of five items measured on a Likert scale ranging from 1 (= strongly disagree) to 5 (= strongly agree) (e.g., “I am worried that my personal data (*such as my online surf and search behavior*, *name*, *and location*) will be abused by others”). We measured *platform attitude* using an adapted version of the *Facebook Attitude Scale* (FAS) (tailoring it to Instagram) that Chua and Chua [71] adapted from the Facebook Questionnaire developed by Ross et al. [72], consisting of seven items measured on a 5-point Likert scale ranging from 1 (= strongly disagree) to 5 (= strongly agree). Two items, “I feel out of touch when I haven’t logged on to Instagram in a while” and “How satisfied are you with Instagram” decreased our indicators for a good fit in a CFA and thus were not included in our analyses. We measured *intended privacy protection behavior* using the scale used in recent microtargeting research by Binder et al. [1], consisting of five items measured on a Likert scale ranging from 1 (= strongly disagree) to 7 (= strongly agree) (e.g., “I will use software that disguises my identity online”). One item “I will deregister from an app or account to protect my data” decreased our indicators for a good fit in a CFA and thus was not included in our analyses. While further evaluating our items for this measurement, we found that the other items concerned changing settings on a platform, using software, or informing oneself. The excluded item might be a very rigorous measure to take, meaning that it could not be as uniformative compared to the rest of the items, which could explain why this item decreased our indicators. In addition, we recognize that the average variance explained for this scale is below the threshold of .50, but we decided not to further alter this scale because other indicators were above their respective thresholds. However, this scale combines various behaviors and does not represent a uniform scale.

**Table 1:** Results of the confirmatory factor analyses.

Measured Constructs	χ^2^	*df*	*p*	CFI	TLI	RMSEA	SRMR	α	ω	AVE
Targeting Knowledge	213.08	2	.008	.99	.98	.09	.01	.90	.90	.69
Perceived Benefits	85.62	5	< .001	.95	.91	.19	.04	.91	.92	.68
Privacy Concerns	27.00	5	< .001	.98	.96	.10	.02	.87	.88	.60
Attitude Towards Instagram	54.09	5	< .001	.94	.88	.15	.04	.83	.83	.50
Intended Privacy Protection Behavior	43.25	5	< .001	.93	.87	.13	.05	.77	.77	.45

Reliability measures are Cronbach’s *α*, McDonald’s ω, and average variance extracted. CFI = Comparative fit index; TLI = Tucker-Lewis index; RMSEA = root-mean-square error of approximation; SRMR = standardized root-mean-square residual; AVE = average variance extracted.

#### Exploratory variable

As an exploratory variable, we measured *algorithmic user agency*. With this construct, we aim to reveal users’ behavior, which is intended to inform the algorithm about their preferences to receive better-tailored content. This means that users consciously interact with certain content in their timelines to see more content like it (e.g., liking a cat video on Instagram to see more cat videos in the future). We included three items: “I like pictures and videos on social media to see more of that content”, “I try to inform the algorithm about myself, to receive better-tailored content”, and “I am selective in what I like or interact with, to let the algorithm know that”. We measured these three items on a Likert scale ranging from 1 (= strongly disagree) to 7 (= strongly agree) (Cronbach’s *α* = .89, McDonald’s ω = .89, *M* = 3.1, *SD* = 1.2, AVE = .73).

### Manipulation check

As a manipulation check, we asked participants if they recalled if there was a disclosure on the Instagram post and what the disclosure stated by asking them to check one of the following statements:

- The Instagram post was a regular post
- The Instagram post was labeled as targeted at me and sponsored
- The Instagram post was labeled as sponsored

## Results

Statistical analyses were conducted using R (version 4.1.2) [65] and jamovi (version 2.0.0.0) [73]. To investigate the differences between the two conditions, we conducted a path model with our mean scores through structural equation modeling in lavaan [74]. The code of our conducted analyses is available on the OSF (https://osf.io/qa2en?view_only=7923aff35ecd44caa90f7c7603912e03). The bivariate correlations, means, and standard deviations for the measured variables are shown in Table 2. For all our hypotheses, we used our SESOI of β = |.15| and an alpha level of .05 as thresholds for acceptance or rejection.

**Table 2:** Means, standard deviations, and bivariate correlations of the measured constructs.

Measured construct	*M* (*SD*)	1	2	3	4	5	6
1 Experimental condition	-	-					
2 Targeting Knowledge	4.1 (1.6)	.27***	-				
3 Perceived Benefits	3.8 (1.5)	.05	.54***	-			
4 Privacy Concerns	4.6 (1.3)	-.05	.03	.00	-		
5 Attitude Towards Instagram	2.8 (0.9)	.02	.03*	.13**	-.04	-	
6 Intended Privacy Protection Behavior	3.7 (1.3)	-.01	.02	.03	.42***	.15**	-
7 Algorithmic User Agency	3.1 (1.2)	.10*	.14**	.21***	-.08	.15**	.00

* *p* < .05,

** *p* < .01,

*** *p* < .001.

Regarding our manipulation check, in our sponsored condition (*n* = 225) 132 participants did not recall a disclosure, 72 participants rightfully recalled a sponsored label, and 21 participants stated the post contained a targeting disclosure. In our targeting disclosure condition (*n* = 225) 189 participants rightfully recalled a targeting disclosure, 19 recalled no disclosure and 17 recalled a sponsored disclosure.

**Fig 1:**
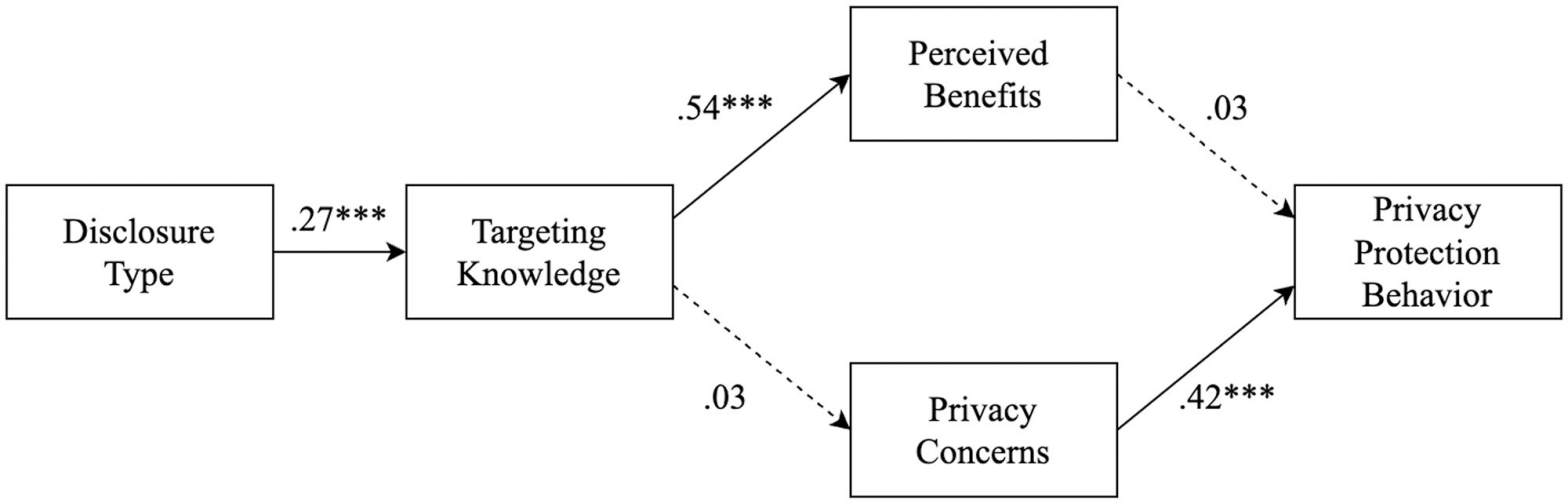
Path model. Numbers represent standardized regression coefficients. Dashed lines indicate a path is either statistically meaningless (not significant) or theoretically meaningless (below β = |.15|). * *p* < .05, ** *p* < .01, *** *p* < .001.

As preregistered, we tested our hypotheses, without our moderation hypotheses, in a path model. Our model fit was evaluated in line with frequently used fit indices [75]. Our model showed an adequate fit: χ2(5) = 8.09, p = .151, χ2/df = 1.62, CFI = 0.99, TLI = 0.98, RMSEA = .04, 90% CI [.00, .08], SRMR = .03. This model is illustrated in Fig 1. Due to our available resources and our maximum sample size, our path model with an inclusion of the moderation would be too complicated and would decrease the power and interpretation of our results. However, we tried to test the model with moderation included, but this led to model fit indices that were far from reasonable to interpret.

Our first hypothesis predicted that exposure to a targeting disclosure would lead to higher levels of targeting knowledge than exposure to a sponsored disclosure. In line with this hypothesis, we found a significant positive effect (β = .27, *p* < .001), leading us to accept H_1_. The second hypothesis predicted a positive relationship between targeting knowledge and perceived benefits. We found a significant positive relationship (β = .54, *p* < .001), supporting this hypothesis. Moreover, through our third hypothesis, we predicted that targeting knowledge would be positively related to privacy concerns, which we found no evidence for in our data (β = .03, *p* = .531), leading us to reject this hypothesis.

Our fifth hypothesis predicted that perceived benefits would be negatively related to intended privacy protection behavior, which we found no evidence for in our data (β = .03, *p* = .471), leading us to reject H_5_. Our sixth hypothesis predicted that privacy concerns would be positively related to intended privacy protection behavior, which we found evidence of in our data (β = .42, *p* < .001), leading us to accept H_6_.

### Moderation

For our fourth hypothesis (H_4_), we proposed that the relationship between targeting knowledge and both perceived benefits (H_4a_) and privacy concerns (H_4b_) would be moderated by users’ attitude towards Instagram. We investigated these hypotheses using the medmod [76] module in jamovi with 1,000 bootstrapped samples. For H_4a_, we did not find a direct relationship between users’ attitude towards Instagram and users’ perceived benefits (*b* = .11, *p* = .088), nor did we find an interaction between users’ attitude and targeting knowledge (*b* = .03, *p* = .441), leading us to reject H_4a_. For H_4b_, the direct effect of users’ attitude towards Instagram was not found to be related to users’ privacy concerns (*b* = -.06, *p* = .450), as was the case for the interaction between users’ attitude and targeting knowledge (*b* = .05, *p* = .279), leading us to reject H_4b_.

### Exploration

Finally, through research question one, we investigate our exploratory variable, algorithmic user agency, and its potential relationship with perceived benefits. Our data showed this relationship (*r* = .21, *p* < .001), meaning that there was indeed a positive correlation between the two constructs.

## Discussion

The current study aimed to investigate whether targeting disclosures on political microtargeted advertisements on Instagram would increase users’ awareness and perceptions of their online privacy, and how this related to their privacy behavior. To do so, we investigated microtargeting disclosures through an integrative path model with a rationale based on the privacy calculus that expected users privacy benefits and costs to be related to increases or decreases in their privacy protection behavior.

We expected that exposure to a targeting disclosure, compared to a sponsored disclosure, would lead to higher levels of targeting knowledge, and thus help users recognize targeted political ads [1, 2, 20]. Although prior research shows mixed results concerning users’ recall and interpretations of disclosures, we found proof of this assumption in our data. One of the consistencies in the existing work on disclosures as a measure to increase transparency is that users do not always recall disclosures correctly [1, 2, 77]. In the current study, we found results that are partially in line with these findings. In our control condition, in which the post was labeled with Instagram’s current sponsored disclosure, 32% of our participants correctly recalled the disclosure. However, in our experimental condition, where we exposed participants to a targeting disclosure based on the COVID-19 misinformation disclosures that Instagram and Facebook used during the pandemic, we found different results. In this group, the vast majority (84%) recalled the targeting disclosure. Although it was not a core question of this study, we, in line with other works, were able to show that the current disclosures regarding sponsored content on Instagram do not work as intended and suffer from a lack of recall and are thus not able to contribute to transparency regarding targeting [1, 19]. On the other hand, we were also able to show that larger, more salient disclosures led to greater recall within our sample, which eventually could lead to greater efficiency of disclosures.

Besides testing the potential effects of our disclosures on targeting knowledge, we expected that users’ awareness of microtargeting and its processes would be positively related to both perceived benefits and perceived privacy risks. We found this relationship only for the perceived benefits. This shows that when users are aware of the advertisement being targeted at them, they have higher levels of perceived benefits, which is in line with work that found more positive evaluations of content when it is personalized [49, 51]. A potential mechanism behind this could be that the fluency of the content being personalized at the user disregards the message being perceived as a warning. While previously disclosures were mainly used to inform or even warn users about content being tailored and therefore more personalized than a ‘regular’ message or advertisement, it could be that users’ are more used to tailoring of timelines on platforms, and even advertisements, that they mainly perceive the benefits of tailoring as a technique and therefore perceive it as a useful or beneficial technique.

Regarding privacy concerns, we did not find a relationship with targeting knowledge. Prior work shows that in the case of behavioral advertising, persuasion knowledge affects privacy risk perceptions. While existing research shows that if people know that they are exposed to personalized messages, this affects privacy risks [54] or that users even feel that these messages are intrusive or creepy, our results did not align with this [23, 53]. Moreover, we would like to point out that our result aligns with the novel work by Dobber and colleagues, who also did not find a relationship between exposure to transparency information and users’ privacy concerns [36]. However, looking at the mean scores for privacy benefits and concerns, we see that the mean for privacy concerns is substantially higher than the mean for perceived benefits, which could imply that even though we did not find a relationship with targeting knowledge, users still have relatively high privacy concerns regarding PMT. A certain privacy threat awareness could have already existed, which might explain why explicitly informing users about the use of their data might not make a difference.

Regarding the relation between targeting knowledge and privacy concerns we would like to point out that based on the fact that we did not find a relation could also, on a speculative basis, be explained by a negativity bias [78]. By default, users might be more focused on the concerns regarding their online privacy, which could explain why there was no relation. In contrast, this might be why we did find a relation between targeting knowledge and perceived benefits, because the benefits of microtargeting might not be the default thing people think about if they are informed about the technique. In addition, a ceiling effect could also limit the potential relationship between targeting knowledge and privacy concerns since the mean score was already relatively high. This might be a threshold that does not increase or decrease by explicitly explaining that users are targeted.

Furthermore, we assumed that the relationship between users’ awareness and perceived benefits and privacy risks would be moderated by users’ attitude towards Instagram, meaning that for users with a more positive attitude towards Instagram, the relation between targeting knowledge and perceived benefits would be stronger and that for those users, the relation between targeting knowledge and privacy concerns would be weaker. Conversely, we expected that, for users with less favorable attitudes towards Instagram, the relationship between targeting knowledge and perceived benefits would be weaker, and the relationship between targeting knowledge and privacy concerns would be stronger. Contrary to existing work, we did not find proof of our moderation hypotheses in our data [58]. One reason for this could be that PMT as a technique is not related to the platform in the perception of users. Even though the implementation of the technique is the same for both Instagram and Facebook, it could be that users’ awareness of tailoring in any form might have risen beyond just the platforms. Tailoring of both content and advertisements is something that happens not only on SNS but also on regular websites and search engines. It is possible that users do not distinguish between tailoring and PMT specifically, and see this separately from the platform they are using.

Moreover, we expected that perceived benefits would be negatively related to intended privacy protection behavior, meaning that users who perceive the benefits of being targeted and receiving personalized advertisements engage less in measures to protect their online privacy. However, we did not find proof for this in our data, which is confirmatory for work on personalization [79] and SNSs [80] (the latter used self-withdrawal, which can be seen as a form of privacy protection behavior). While we made the assumption based on the privacy calculus, we do recognize other work that found no relation between perceived benefits and users’ desire to protect their privacy online. It is possible that benefits and privacy protection behavior are rather independent constructs and that privacy protection behavior might not be just the outcome of a calculation that users make between benefits and risks [43, 60].

Furthermore, we assumed that privacy concerns would be positively related to intended privacy protection behavior, which we indeed found proof for in our data, consistent with other work regarding social media [43, 60] and microtargeting [1, 46]. However, we would like to emphasize that this does not show a calculation or weighing between privacy concerns and perceived benefits. This calculation could be visible in direct relations or interactions between the two constructs, or in the perceived benefits being larger than privacy concerns, which is not the case in the current study. The fact that we did find a relationship between privacy concerns and protection behavior but did not find a relationship between benefits and protection behavior would not necessarily imply that users weigh the factors beforehand. This mainly shows that when users have more privacy concerns, independent of whether they perceive the targeted ad to be beneficial or not, they aim to protect themselves online.

Additionally, as an exploratory research question, we investigated the relationship between users’ perceived benefits and algorithmic user agency [63]. This means that users are aware of the algorithmic processes taking place (i.e., receiving content because the algorithm ‘knows’ that you interacted with content like that before), and try to actively control these processes by consciously interacting with content, which could be perceived as a potential proxy for their willingness to actively use algorithms to their advantage. We found a correlation between perceived benefits and algorithmic user agency. In addition, even though they were smaller, we also found correlations between the construct and our manipulation, targeting knowledge and attitudes towards the platform. This could imply that users who are explicitly informed about targeting practices might make use of their agency, but also that users with higher levels of knowledge about targeting and users with more favorable attitudes towards the platforms might operate in the same way. However, we emphasize that these findings are correlations and that we do not attempt to make causal claims regarding this subject.

Moreover, we argue that algorithmic user agency might fit as a secondary outcome of the calculus rationale for PMT. Users who experience higher benefits might interact with the advertised content and the algorithm more than users who are more focused on the risks, which in turn might lead them to protect their online privacy. Finally, although algorithmic user agency is a newly developed construct and, to the best of our knowledge, the first time this has been empirically investigated in an experiment, we encourage other researchers to build on these findings and explore users’ behaviors and potential interactions with algorithms regarding PMT.

### Limitations and future work

To avoid influencing participants beyond the scope of our experiment, we created a political party that supposedly posted the Instagram content in our stimulus material. However, we recognize that political behavior and, as an extension, voting are behaviors that are complex and do not change within a moment of looking at an Instagram post. While avoiding ethical risks, we also recognize that a novelty effect might have taken place for participants. It is not possible that participants ever saw content from this political party, which might have led them to focus more on the Instagram post and less on the disclosure, potentially underestimating the power of the effect we found. On the other hand, we forced participants to view the post, including the disclosure, for a minimum of 15 seconds, which might have led to an overestimation of not only the effect of the disclosure on targeting knowledge, but also on the percentage of participants who correctly recalled the disclosures in our sample, which is remarkably higher than in other studies. We believe that the field of personalization and microtargeting research will benefit from large-scale field experiments with actual usage data gathered through web scraping or even data donations.

Concerning the statements used in our stimuli, even though we used them to improve ecological validity since they were used in the German Wahl-o-mat, we acknowledge that the statements are not mutually exclusive and that participants who agree with a pro-climate change regulations statement could also agree with an anti-climate change statement. After investigating these subgroups, however, we found that only 42 participants were exposed to the anti-climate change regulations statement (21 per condition). Therefore, we did not perform our analyses with separate subgroups. Future research could benefit from statements that are mutually exclusive, as this could make for better comparisons.

Regarding the effect of our manipulation on targeting knowledge, we would like to point out that participants saw our manipulations in a controlled experimental setting (i.e., a timer that tried to force them to look at the Instagram post for 15 seconds). This might have led them to use not only more cognitive resources to process the message, but also the disclosure itself, compared to a less superficial setting. In a setting with higher ecological validity, SNS users might be exposed to more messages in a shorter period, which leads to the question of whether they would process the message, and accordingly the disclosure, less central and more peripheral, as explained in the Elaboration Likelihood Model [52]. In a real-world setting, users might focus more on the actual content before focusing on a disclosure. Earlier work, for example, showed that users focus on a video or image in their timelines before they focus on anything else [81].

In the current study, our aim was to simulate microtargeting by asking participants if they agreed with statements concerning climate change regulations. Even though we showed our participants’ captions on the Instagram post that we assumed fit their views more than a general statement would, we recognize that there is a chance that this was not the case, and the post might not have been perceived as microtargeted. In addition, we recognize that there is a chance that participants were aware that the post we showed them was not actually based on their personal data. Besides, there are many different regulations regarding climate change reduction, and people might generally agree with the regulations but could also disagree with the ones we selected (e.g., the speed limit on the German highway). However, we would like to point out that in a real-world setting, ads that users receive might not always be perceived as perfectly fitting, even though this is what senders try to achieve.

We believe that the field of disclosure research, as well as legislators and governmental institutions, will benefit from research that includes a more practical approach and not only investigates the effects and relations regarding disclosures, but also the design of the disclosures themselves and which designs are easier recalled by users. Recallment is a large part of disclosures effectiveness, as disclosures need to be recalled and perceived to inform people. We also want to emphasize that research would benefit from investigating whether these disclosures are perceived as annoying or disturbing for users. Besides, from a policy and transparency perspective, future research would benefit from a clearer investigation of why users recall (or do not) certain disclosures, which, for instance, might be due to a lack of exposure but could also have to do with a lack of interest in the information about an ad, if an ad is directly recognized as such, which might lead to the user directly scrolling to other content. We believe that eye-tracking studies combined with more ecologically valid experiments where users scroll through the timelines of their own SNS accounts might provide clearer insights into user behavior on SNS and the effectiveness of disclosures if they are embedded on the platforms.

Regarding our moderation hypothesis, we would like to emphasize that to detect potential moderation, this study might have had a sample size that was not sufficient to detect moderation with sufficient power [82]. Finally, apart from the effect of our manipulation on targeting knowledge, this study has a cross-sectional design and, therefore, does not allow us to investigate causal relations, meaning that other paths in our model are bidirectional.

## Conclusion

The targeting of political advertising on social media is something that users do not see occurring in the foreground, or recognize at all. However, governments, regulators, and researchers have reached consensus on the need to improve transparency. The current study investigated the effectiveness of targeting disclosures as a means to improve transparency, and subsequently, scrutinizes users’ perceptions of their online privacy based on the privacy calculus. In an integrative model, we found that our disclosure affected users’ targeting knowledge, which was positively related to their perceived benefits of PMT. Nevertheless, we did not find a relationship between targeting knowledge and users’ privacy concerns regarding PMT. Additionally, neither of these relationships was moderated by users’ attitudes towards the platform they were using. In addition, we did not find a relationship between users’ perceived benefits and intended privacy protection behavior; however, we did find a relationship between privacy concerns and intended protection behavior. Together, our findings show that if users are alerted about targeting practices taking place on platforms, they see the benefits of personalization, and that this does not relate to behavior that protects their privacy. In addition, we see that only if users view personalization as a privacy violation they might engage in behavior to protect their privacy. Although the exact role of targeting disclosures and their desired designs may still be a topic of debate for legislators, this study provides a first interpretation of what these disclosures mean to users’ privacy perception if they are made aware that they are microtargeted.
